# Cigarette Taxes and Smoking Participation: Evidence from Recent Tax Increases in Canada

**DOI:** 10.3390/ijerph8051583

**Published:** 2011-05-16

**Authors:** Sunday Azagba, Mesbah Sharaf

**Affiliations:** Department of Economics, Concordia University, 1455 de Maisonneuve Blvd. West, Montréal, Quebec, H3G 1M8, Canada; E-Mail: m_shara@live.concordia.ca

**Keywords:** cigarette taxes, smoking participation, elasticity, socio-demographic factors

## Abstract

Using the Canadian National Population Health Survey and the recent tax variation across Canadian provinces, this paper examines the impact of cigarette taxes on smoking participation. Consistent with the literature, we find evidence of a heterogeneous response to cigarette taxes among different groups of smokers. Contrary to most studies, we find that the middle age group—which constitutes the largest fraction of smokers in our sample—is largely unresponsive to taxes. While cigarette taxes remain popular with policy makers as an anti-smoking measure, identifying the socio-demographic characteristics of smokers who respond differentially to tax increase will help in designing appropriate supplementary measures to reduce smoking.

## 1. Introduction

It is well established that tobacco use is a major cause of preventable morbidity and mortality in the World. The World Health Organization (WHO) [1] links five million deaths each year to tobacco use and by 2030 tobacco related deaths are estimated to be eight million yearly. Several studies have documented the health consequences of smoking; these include cardiovascular disease, cancer and chronic obstructive pulmonary disease (emphysema and bronchitis). The average life span of a smoker is reduced by 6 to 10 years [2,3]. The substantial social, economic and health costs induced by tobacco use have led many countries to adopt higher cigarette taxes as a policy to reduce smoking.

While the effectiveness of cigarette taxes depends on how smokers respond to such tax increases, the literature mostly agrees that cigarette taxes are in general effective, with some exceptions. Empirical evidence shows that certain socio-demographic groups of smokers may be less tax-responsive than others, e.g., [4,5]. Identifying the socio-demographic characteristics of smokers who respond differentially to tax increase will help in designing additional measures to reduce smoking. For example, for smokers with a severe self-control problem, higher cigarette taxes may only reduce their monetary well-being, without affecting their smoking behavior. Evidence about participation elasticities in the literature is mixed, though there is a consensus on the existence of a differential response to cigarette tax increase. Fletcher *et al.* [6] use data on adolescents from the National Longitudinal Study of Adolescent Health and find evidence of heterogeneous price elasticities for tobacco use across adolescent groups. Using a latent class framework, these authors find that a particular group (heavy smokers) is unresponsive to cigarette taxes.

A conventional belief among academics and policy makers is the notion that young smokers are more responsive to cigarette prices. This notion is grounded on the following: peer pressure effects, experience of smoking [7], the short-sighted attitude of the young, the larger ratio of smoking expenditure to disposable income for the young versus the old [8]. In an early study, Lewit and Coate [9], using data from the 1976 National Health Interview Survey, find a larger participation elasticity for young adults than for adults over 35 years. Additional support for the inverse relationship between elasticity and age has been documented by some recent studies [4,10–13] (for a comprehensive review, see Chaloupka and Warner [14]). Using multiple cigarette price measures, Ross and Chaloupka [15] find that higher cigarette prices reduce smoking participation among high school students. They also find some negative price effects on smoking intensity. In a recent US study, Carpenter and Cook [16] find a negative and significant tax effect on youth smoking participation.

The results of some other studies run contrary to the general belief that the young are responsive to cigarette costs. Chaloupka [17] find no significant price impact on young adults (ages 17–24) and highly educated individuals. Furthermore, he finds that individuals aged 25–64 show a significant long-run response to a change in price. Wasserman *et al.* [18] find that the elasticity estimates are sensitive to the inclusion of an index for smoking restriction. They find that young smokers are not sensitive to price increases when the restriction index is added to their cigarette demand model. Using the onset of smoking and discrete-time hazard models, DeCicca *et al.* [19] find marginal tax effects on youth smoking behavior. Even on theoretical grounds, the relation between age and cigarette demand elasticity cannot be determined a priori as there are a number of interacting and offsetting influences that affect smoking responses [19]. This implies that the differential impact of tax increases on cigarette participation by age is an empirical issue.

Another empirical regularity (*c.f.* Gospodinov and Irvine [20]) is that cigarette demand is relatively more elastic for low educated/income smokers than high educated/income smokers [5,21]. Gruber and Koszegi [21] show that individuals in the lowest income quartiles are most sensitive to cigarette prices and those in the highest quartile are least sensitive. They also find differences by education groups, with higher elasticities for low education groups. The authors also suggest that an optimal tax would range from $5 to $10 (see Coleman and Remler [22] for a view on equity and fairness issues). Townsend *et al.* [5] find similar results using data from the British general household survey. They find that smokers in lower socioeconomic groups are more price-responsive than those in higher socioeconomic groups. With respect to tax-response by gender, several studies find that men are more responsive to cigarette taxes than women, e.g., [9,23–25], while other studies find the reverse [4,26]. Stehr [26] showed that most US studies who find that men are more responsive to cigarette taxes than women failed to control for state-specific gender gaps in smoking rates that are correlated with cigarette taxes. He finds that women are twice as responsive to cigarette taxes as are men after controlling for gender-specific state fixed effects.

The extant literature on the relationship between cigarette costs and smoking behavior has been largely US focused. Most of these studies use low cigarette prices from the post Master Settlement Agreement (MSA) era, which depend on cross-state variation. Consequently, cigarette tax/price estimates may reflect unobserved state-specific sentiment toward smoking. The anti-smoking sentiment may be reflected in a state’s taxes, for example tobacco producing states in the US may charge lower taxes. However, while this anti-smoking sentiment may be less of a concern in Canada given that there is no major tobacco producing province (Gopodinov and Irvine [27]), this study controls for provincial fixed effects. For a review of the different ways to account for state anti-smoking sentiment, see Carpenter and Cook [16].

The objective of this paper is to examine the impact of the recent upward trend in Canadian cigarette taxes on smoking participation. This study uses longitudinal data from the National Population Health Survey (NPHS) 1998/99–2008/09. The analysis controls for individual contextual factors, unobserved heterogeneity and other variables that influence smoking behavior. The use of this individual level data allows us to examine heterogeneous tax effects on the smoking participation of various population subgroups. We stratify individuals by key socio-demographic factors. To avoid having biased estimates of the impact of taxes on smoking participation, we account for inter-provincial differences in cigarette taxes.

This paper contributes to the literature in several dimensions. First, using recent tax data provides an update on the efficacy of cigarette taxes in altering smoking behavior. Relying on elasticity estimates obtained during periods of low prices may be of limited use, given that behavioral responses are likely to evolve over time. Second, using longitudinal data enables the long-term impact of taxes to be studied. Also, observing individuals over a longer period inevitably lead to a better estimate of behavior than cross-sectional analysis.

The structure of this paper is as follows: in Section 2 we present a brief background on the theoretical literature and cigarette taxes in Canada. Section 3 describes the data and methodology. Section 4 presents the results and conclusions are provided in Section 5.

## 2. Brief Background Literature

Economists have formulated models to explain the rationale for addictive consumption. The general point of reference is the rational addiction (RA) model of Becker and Murphy (BM) [28]. In this model, consumers optimally make smoking decisions with knowledge of the health consequences of tobacco use, the addictive nature of cigarette smoking and all the monetary costs. Government intervention through higher taxes will necessarily make a smoker worse off in the BM model. A central assumption of the RA framework is time consistency, that is to say, future preferences coincide with the current decision to smoke.

In contrast to the time consistent preferences in the RA model, the behavioral economics literature uses hyperbolic discounting to characterize consumers’ preferences for addictive goods as time inconsistent. O’Donoghue and Rabin [29] describe time inconsistent preferences as ‘present-biased preferences’ Smokers in this framework place a higher value to immediate gratification, hence, significantly discount the long-term negative impact. O’Donoghue and Rabin [29,30], and Gruber and Koszegi [31] show how time-inconsistent behavior depends on perceived future beliefs of self-control. Naive agents tend to overestimate their ability to control future behavior while sophisticated agents fully understand future self-control problems. Due to the incentive effect, sophisticated smokers are more likely to refrain from smoking than naive smokers. Incentive effect here refers to a situation where sophisticated smokers refrain from current consumption in order to prevent future indulgence, see O’Donoghue and Rabin [30] for details.

Gruber and Koszegi [31] suggest that if smokers are sophisticated about their self control problems and responsive to prices, taxes could act as a self-control device for them. They suggest that government intervention in the tobacco market should not be limited to externalities (costs that smokers impose on others) but should also include smoking internalities. Self control and failure to attain a desired future level of smoking are the two key features that separate time-consistent from time-inconsistent agents.

Hersch [32] argues that smokers’ support for government regulations that restrict smoking in public areas is an indication of a lack of self control among smokers. Providing further support of the time inconsistent smoking model, Gruber and Mullainathan [33] find that cigarette taxes can increase the well-being of likely smokers. Bernheim and Rangel [34,35] argue that addictive goods can sometimes interfere with the decision process of the brain, and lead to wrong ‘cue-conditioned’ cravings. Using taxes when consumption of addictive goods is driven by cues may be counterproductive. The distributional burden of cigarette taxes will be regressive, if low income smokers have small short term discount factors, and progressive, if their long term discount factor is smaller [22].

### Cigarette Taxes in Canada

In Canada, cigarettes are taxed at both the federal and provincial levels. A key feature of the Canadian tax system is that there is a substantial degree of variation in cigarette taxes across provinces. In February 1994, cigarette taxes were subject to a substantial reduction of about 50% by the federal government and five of the Eastern provinces, in an attempt to fight smuggling of cigarettes (for details, see Gruber *et al.* [36]). The Western provinces decided to keep taxes constant, and to fight smuggling in other ways. The cigarette tax in Canada was fairly stable across provinces between 1995–2000.

The Federal Tobacco Control Strategy (FTCS) was launched by the federal government in April 2001, with four strategic components: protection, prevention, and cessation and harm reduction. Subsequently, cigarettes were subject to a series of tax increases as a major instrument to achieve the objectives of this strategy.

The first tax increase was applied in April 2001, raising the federal excise tax to $10.65 per carton. In May 2001, the federal excise tax was further increased to $10.99 per carton, and by July 2002 it reached $15.85 per carton. Since 2002, there has been a steady small increase in the nominal excise tax to offset the impact of inflation on the real federal excise tax. The increase in the federal tax was accompanied by increases at the provincial level, but with different magnitudes. Table 1 shows the average real taxes by provinces between 1998 and 2008 [37].

## 3. Data Description and Variables

The data for this study come from the Statistics Canada NPHS household component. NPHS is a nationally representative sample of the Canadian population which collects vital information on health related behavior, as well as corresponding economic and social-demographic variables. The survey excludes those living on Indian Reserves and Crown Lands, full-time members of the Canadian Forces Bases and some remote areas of Ontario and Quebec.

The NPHS commenced in 1994/95 with a subsequent follow up every two years. Since the first cycle, there have been seven follow-up surveys, and cycle 8 (2008/09) is currently available. The first cycle contains responses from 17,276 individuals. NPHS became strictly longitudinal from cycle 4 (2000/01) and the first three cycles (1994/95, 1996/97 and 1998/99) have both cross-sectional and longitudinal components. This study uses data from cycle three (1998/99) to cycle eight (2008/09).

The dependent variable, smoking participation, includes daily and occasional smokers. We restrict the sample to those 12–65 years due to a potential contamination of the analysis. The smoking prevalence of the older age cohort is relatively small for this group (>65 years) and also their health related issues may further complicate the analysis. This study does not examine the intensity (number of cigarettes) of smoking, though this is available in the NPHS data set. Adda and Cornaglia [38] argue that intensity of smoking should be in terms of the cotinine intake level not the number of cigarettes smoked. These authors find that smokers exhibit compensatory behavior by reducing quantity but extracting more cotinine in response to cigarette tax increase. However, NPHS does not have the cotinine level of smokers.

Cigarette taxes are used instead of prices since the former ‘is a more exogenous measure’ than the latter [39]. NPHS does not collect data on cigarette taxes. Historical tax data are obtained from the respective provincial tax offices. The tax rates are matched with each respondent’s province of residence and date of interview available in the NPHS. To obtain the real cigarette tax per carton, both the federal and provincial consumer price index obtained from CANSIM are used to deflate each of the nominal tax components.The sum of the deflated taxes is the real exercise tax in 2000 dollars.

In addition to the cigarette tax, this study follows standard practice in the tobacco literature by using a number of control variables. Age has three categories: 12–24, 25–44, and 45–65 (reference category). Household income is represented by three dummy variables: low income (reference category), middle income, and high income. Gender is captured by a dummy variable (male = 1, female = 0). Household size is family size. Studies show that macroeconomic situations can affect smoking decisions [40,41]. The macroeconomic environment is captured by a dummy variable (employed = 1, unemployed = 0) and the provincial unemployment rate. Four dummy variables represent individual educational attainment: less than secondary (reference category), secondary, some post secondary, and post secondary. Marital status is represented by three dummy variables: married, separated, and single (reference category). Ethnicity is captured by a dummy variable (immigrant = 1, Canadian born = 0). Provincial dummy variables are included with British Colombia as the reference category. Health status is not included in the model due to a potential endogeneity (reverse causality) issue between the smoking decision and health status. Table 2 provides a complete definition of the variables used in this analysis.

### Empirical Strategy

The empirical analysis is based on the following reduced form probit specification:

(1) $$𝒫𝓇\;({𝒮}_{𝒾𝒿𝓉}=1)=\Phi (\theta +\gamma {\text{Cigarette}\;\text{tax}}_{𝒿𝓉}+\beta \prime X_{𝒾𝓉}+{𝒯}_{𝓉}+{𝒬}_{𝒿𝓉}+\alpha_{𝒾}+{ɛ}_{𝒾𝒿𝓉}$$

where *i* indicates the individual, *j* represents province of residence, and *t* represents the year, *𝒮* represents smoking participation, ***𝑿*** is a vector of other control variables including: age, income, gender, household size, employment status, education, marital status and ethnicity. *𝒯* captures the time trend of smoking behavior, the province fixed-effect variable, *𝒬*, is included to capture regional smoking ban regulations and other cultural factors that may be region-specific. In Canada, the municipal Act 2001 empowers municipalities to control smoking in public places. *α_𝒾_* represents time invariant individual-specific heterogeneity and *ε _𝒾𝒿_* is the standard time variant residual term which is adjusted for clustering at the individual level. The main coefficient of interest *γ* represents the impact of cigarette taxes on smoking participation.

To allow unobserved heterogeneity to be correlated with observed covariates, the unobserved heterogeneity is parameterized using the Mundlak [42] device, a parsimonious adaptation of Chamberlain’s [43] random effects probit model. Some recent studies that have used the Mundlak approach to account for correlated random effects include: Contoyannis and Li [44], Mentzakis [45] and Kjellsson *et al.* [46]. The unobserved effects are parameterized as:

(2) $$\alpha_{𝒾}=\psi +{\bar{\chi }}_{𝒾}\xi +{𝓊}_{𝒾}$$

where *χ̂ _𝒾_* controls for unobserved heterogeneity and is the within-individual means of time varying covariates. Variables included in *χ̂ _𝒾_* are listed in Table A1. *𝓊_𝒾_* is assumed independent of *𝓍_𝒾_* and distributed $𝒩\;(0,\sigma_{𝓍}^{2})$. Substituting Equation (2) into Equation (1) depicts the effect of changing *𝓍_𝒾𝒿_* but holding the time average fixed.

## 4. Descriptive Statistics

The average smoking prevalence by selected groups from 1998–2008 is reported in Table 3. The NPHS data used for this study contains 56,770 observations, after excluding missing observations. Generally, the average prevalence rate in Canada has been declining for more than two decades. Table 3 shows that the percentage of smokers is lower for those who are females, married, older, with high income, and more education.

**Figure 2.**
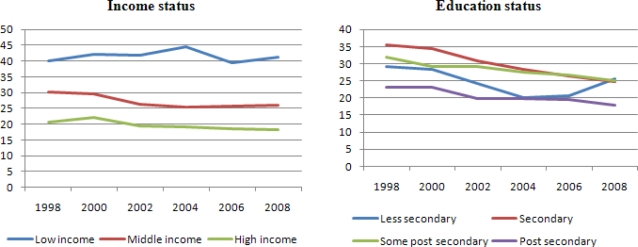

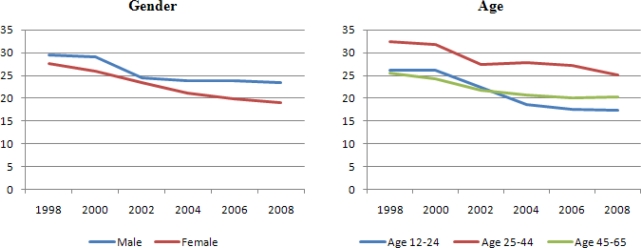
Smoking participation by selected characteristics. Source: These figures are based on Table 1.

The decreased proportion of Canadian smokers is larger for most of the selected groups between the years 2000 and 2002 and average real cigarette tax went up during this period (see Table 1 and Figure 2). It should be noted that graphic pictorial warning labels were introduced during this period in Canada. However, some studies show that pictorial warnings have negligible impact on smoking prevalence in Canada [27,47]. There is a large percentage tax increase between 1998 and 2008 across all Canadian provinces. This tax increase is more than 100% for all of the eastern provinces (Prince Edward Island, Nova Scotia, New Brunswick, Quebec, and Ontario) that had about a 50% tax reduction in 1994. An interesting observation from Tables 1 and 3 is that the provinces of Newfoundland and British Columbia had the lowest percentage tax increase between 2000 and 2002; as well as corresponding smallest smoking prevalence decrease. It should be noted that cigarette taxes were already at high levels in these areas; unlike other provinces, the tax change in Newfoundland and British Columbia did not have a large effect on smokers because they were already tax sensitized with the caveat that cigarette taxes caused the decline.

### 4.1. Estimation Results

The smoking participation estimates are presented in Table A2. For brevity, we present results for the full model only for the overall sample and for relevant variables. However, the full model is available upon request. The results confirm the standard socioeconomic (SES) gradient in cigarette smoking with respect to the income variables. The higher and middle income groups are less likely to be smokers than the low income group. The education variables show some SES gradient. In particular, individuals with post secondary education are less likely to smoke than those with less than secondary education.

The effect of marital status on smoking prevalence is negative just as the unconditional data in Table 3 suggest. The size of the household negatively affects smoking prevalence. Marriage and household size effects affirm the relevance of family setting on the smoking decision. The positive sign of gender variable confirms the standard results that males are more likely to be smokers. Age has a significant and negative impact on smoking participation. Some of the provincial dummies are significant. This shows that it is important to control for unobserved provincial factors that affect cigarette smoking.

### 4.2. Cigarette Tax Results

Since a large part of the cigarette tax is determined at the provincial level, we suspect there may be an identification issue with cigarette taxes when province dummy variables and year trend are included in the model. As a simple way of assessing the within-province variation in cigarette taxes over the data period, a variance inflation factor (VIF) of 6 (R^2^ = 0.8334) is obtained when cigarette tax is regressed on provincial dummies and trend. The VIF implies there is sufficient within-province variation in cigarette taxes over the sample period.

The key policy variable, real cigarette tax, has a negative and significant impact on smoking participation. Since the estimated coefficients from the probit model provide no quantitative value, the average partial effect and tax elasticity are also reported. Here and in what follows, our interpretation will focus on the elasticity estimates. The tax elasticity estimate for the whole population is −0.227. This result implies that if there is a 10% increase in taxes then smoking participation will fall by about 2.3%. While this result shows that tax increases entail a modest reduction in smoking participation, this finding may not be a generalized response outcome for all socio-demographic groups. Though, not directly comparable, Sen and Wirjanto [48] examine the impact of the cigarette tax decreases that occurred in 1994 on Canadian youth smoking behavior. They find participation elasticities between −0.10 and −0.14, and initiation/persistence elasticities between −0.2 and −0.5. In the next section, we examine the heterogeneous tax responses of key socio-demographic groups that have been examined in the extant literature.

### 4.3. Heterogeneous Responses

The results in Table 4 present differential tax responses by gender, household income and self-rated health status. We find that the participation tax elasticity is numerically larger and significant for males. The elasticity for males and females are −0.322 and −0.120 respectively. As a rule-of-thumb to assess whether the response to tax varies significantly by gender, income, education, age and health status, we estimate a pooled model that includes the cigarette tax interacted with dummy variables for these groups. We find a significant difference for all groups except income and health status. Due to the small sample size of the low income group, we group income into two categories: low income category represents individuals in the low/middle income household and high income group are individuals in the high income household. We find that the low income group is more responsive to taxes than the high income group. While the participation tax elasticity of the high income group (−0.202) is larger than the low income group (−0.183), it is not statistically significant. Based on self-reported health status, we group individuals into two categories: low health if individuals report good/fair/poor health and high health if individuals report excellent/very-good health. We find that the high health group is more tax responsive than the low health group. While the health status is self assessed, maybe the high health individuals care more about their functional health.

Following DeCicca and McLeod [40], we group formal education into two groups: low education represents individuals with secondary education or less (high school or less) and high education is defined otherwise (greater than high school). As expected, the low educated group is more tax sensitive with an elasticity of about −0.41 while higher educated individuals are far less sensitive with an elasticity of −0.03. Though not reported, we find a tax elasticity of −0.555 for low education group and −0.070 for high education group when low education is defined as individuals who did not complete secondary education (high school). However, the estimation for two categories only, may mask a more revealing tax effects. We estimate the tax effects for the four different education categories: less secondary, secondary, some post secondary, and post secondary (see Table 5).

The estimated tax elasticities for each category are as follows: less secondary (−0.555), secondary (−0.218), some post secondary (−0.018) and post secondary (−0.042). To study the heterogeneous tax effects across age groups (results reported in Table 6), we group individuals into three age categories: 12–24, 25–44 and 45–65. We find that those aged 45–65 are more responsive to cigarette tax than those aged 12–24 and 24–44. In recent studies, using data from Youth Risk Behavior Survey (1991–2005) Carpenter and Cook [16] find smoking participation elasticities between −0.2 and −0.5. Tauras [49] examine the impact of smoke-free laws and cigarette prices on adult cigarette demand. He finds a participation price elasticity of −0.126. The participation tax elasticity estimates for age 45–65 group, −0.240, is twice as large as that for age 12–24, −0.122; and 25–44, −0.114. These results are somewhat different from Sloan and Trogdon [11]. The authors use data from the Behavioral Risk Factor Surveillance System (1990–2002) to examine the impact of the Master Settlement Agreement on cigarette consumption for adults aged 18 and older. Though, Sloan and Trogdon [11] find that participation elasticity decreases with age, the estimates were not different for those aged 25–44, −0.10 and 45–64, −0.10. They find significant elasticity estimates only for aged 18–20, −0.27; and 25–44, −0.10. We re-estimate our model using two age categories: 18–40 and 41–65 and the results in Table 6 show that the age group 41–65 (−0.296) is more tax responsive than age group 18–40 (−0.015). These results suggest that the age group 25–40 is the least tax responsive group. Though not reported, we find the participation tax elasticity for aged 25–40 to be −0.075 (this is smaller for those aged 25–44 reported in Table 5 column 3).

As a further robustness check, we re-estimate our model using data from 1998 to 2002 (see Table 7). The largest tax change occurred in most of the Canadian provinces between 2000 and 2002 (see Table 1). The smoking participation rate also witnessed a greater fall during this period (see Figure 2). We hypothesize that the tax impact will be higher for this period. However, as expected the tax effects increased in all the model specifications with the exception of the age category, 25–44 which remain unresponsive.

## 5. Conclusions

In this paper, we examine the impact of the recent upward trend in Canadian cigarette taxes on smoking participation. This study uses longitudinal data from the confidential National Population Health Survey (1998–2008). The panel structure of this data set enables us to estimate the long-term response to tax changes as well as controlling for province fixed effects and unobserved individual heterogeneity. We find that the tax elasticity estimate for the whole population is −0.23. This means that if taxes increase by 10%, smoking participation will fall by about 2.3%. This result is consistent with a recent study (Sen and Wirjanto [48]). While this result shows that tax increases led to a modest reduction in smoking participation, this finding may not be a generalized response outcome for all socio-demographic groups. The results of this study indicate that higher cigarette taxes have a differential impact on smoking participation across different groups of smokers.

We find that the participation tax elasticity is numerically larger and significant for males. The elasticities for males and females are −0.322 and −0.120 respectively. This finding is consistent with previous studies who find that men are more responsive to cigarette taxes than women e.g., [9,23–25], In line with most previous studies, e.g., [5,22], we find that the low income group is more responsive to taxes. The participation elasticity is not statistically significant for the high income group. Analogously, the low educated group is more tax sensitive than the high educated group. The differential response of low income/education smokers versus high income/education smokers raises the debate about the distributional impact of such taxes. While this issue remains contentious, our findings do not aim to resolve it, as it is beyond the scope of this study.

The literature mostly agrees that cigarette taxes are in general effective, with some exceptions. Contrary to most studies, we find that the middle age group—which constitutes the largest fraction of smokers in our sample—is largely unresponsive to taxes. While cigarette taxes remain popular with policy makers as a key anti-smoking measure, their effectiveness largely depends on how people respond to them. Identifying the socio-demographic characteristics of smokers who respond differentially to tax increases will help in designing appropriate supplementary measures to reduce smoking as there is no a “one-size fits all” strategy for discouraging smoking [24].

## Figures and Tables

**Figure 1.**
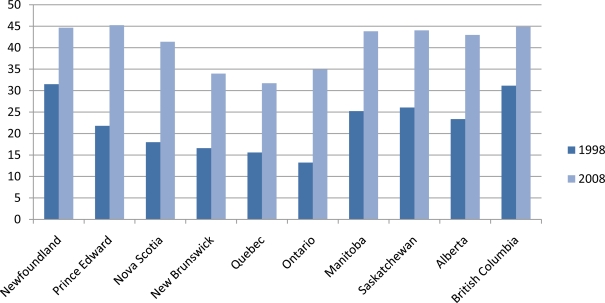
Average real cigarettes tax in Canada by province.

**Table 1.** Average real cigarettes tax (in 2000 dollars) per carton for each Canadian province.

	
	**1998**	**2000**	**2002**	**2004**	**2006**	**2008**
Newfoundland	30.15	29.44	38.24	41.62	43.25	42.74
Prince Edward	20.21	20.46	33.18	41.29	40.23	41.80
Nova Scotia	17.15	17.54	32.41	39.63	38.74	39.43
New Brunswick	15.85	16.28	30.05	34.12	32.76	32.41
Quebec	14.95	16.64	29.02	31.98	30.72	30.44
Ontario	12.65	13.42	26.61	32.27	32.79	33.35
Manitoba	24.15	24.46	38.06	43.63	42.82	41.94
Saskatchewan	24.95	24.85	40.05	43.47	42.51	42.15
Alberta	22.15	21.59	39.00	40.78	38.68	40.74
British Columbia	30.15	29.76	41.28	45.91	44.20	43.38

Source: Provincial Department of Finance and authors’ calculations.

**Table 2.** Descriptive statistics.

**Variable**	**Mean**	**S.D**
Smoking	0.245	0.0018
Cigarette tax	30.350	0.0416
Trend (*𝒯*)	5.060	0.0143
Age	37.870	0.0630
Low_income	0.063	0.0010
Mid_income	0.455	0.0020
High_income	0.354	0.0020
Male	0.500	0.0021
Female	0.500	0.0021
Household size	3.200	0.0059
Employed	0.688	0.0019
Unemployed	0.227	0.0018
Unemployment rate	7.260	0.0086
Less_ secondary	0.231	0.0018
Secondary	0.125	0.0013
Some post secondary	0.256	0.0018
Post secondary	0.362	0.0020
Married	0.551	0.0021
Separated	0.095	0.0012
Single	0.320	0.0020
Canadian	0.852	0.0014
Immigrant	0.146	0.0015
Newfoundland	0.019	0.0005
Prince Edward	0.005	0.0003
Nova Scotia	0.032	0.0007
New Brunswick	0.026	0.0006
Quebec	0.255	0.0018
Ontario	0.368	0.0020
Manitoba	0.036	0.0008
Saskatchewan	0.033	0.0007
Alberta	0.106	0.0013
British Columbia	0.119	0.0014
*N*	*56770*	

The statistics are weighted using the NPHS sampling weights.

**Table 3.** Selected characteristics of smoking participation (in %) at each cycle, aged 12–65.

	
	**1998**	**2000**	**2002**	**2004**	**2006**	**2008**	**Overall**
Whole sample	28.6 (0.4)	27.4 (0.4)	24.0 (0.4)	22.5 (0.4)	21.8 (0.4)	21.2 (0.4)	24.5 (0.2)
Male	29.6 (0.6)	29.0 (0.6)	24.4 (0.6)	23.8 (0.6)	23.8 (0.6)	23.4 (0.7)	25.8 (0.3)
Female	27.6 (0.6)	26.0 (0.6)	23.5 (0.6)	21.2 (0.6)	19.9 (0.6)	19.1 (0.6)	27.6 (0.6)
Age 12–24	26.1 (0.1)	26.1 (0.9)	22.4 (1.0)	18.6 (0.9)	17.5 (0.9)	17.3 (1.1)	21.6 (0.4)
Age 25–44	32.5 (0.6)	31.7 (0.6)	27.4 (0.7)	27.8 (0.7)	27.1 (0.7)	25.1 (0.8)	28.8 (0.3)
Age 45–65	25.5 (0.7)	24.3 (0.7)	21.8 (0.6)	20.8 (0.6)	20.0 (0.6)	20.3 (0.6)	22.1 (0.3)
Less secondary	29.2 (0.8)	28.4 (0.8)	24.1 (0.9)	20.2 (0.9)	20.7 (1.0)	25.6 (1.2)	32.0 (0.4)
Secondary	35.6 (1.2)	34.5 (1.3)	30.8 (1.3)	28.4 (1.4)	26.5 (1.4)	24.7 (1.4)	30.5 (0.5)
Some post secondary	32.0 (0.8)	29.3 (0.8)	29.3 (0.9)	27.6 (0.9)	26.8 (0.9)	25.1 (1.0)	28.6 (0.4)
Post secondary	23.0 (0.7)	23.0 (0.7)	19.7 (0.6)	19.8 (0.7)	19.6 (0.7)	18.0 (0.7)	20.5 (0.3)
Newfoundland	30.2 (1.7)	27.0 (1.7)	26.4 (1.8)	24.6 (1.8)	20.3 (1.7)	20.0 (1.9)	25.1 (0.7)
Prince Edward	38.1 (1.9)	34.5 (2.0)	28.0 (1.9)	26.2 (2.0)	22.5 (1.8)	18.3 (1.9)	28.5 (0.8)
Nova Scotia	32.1 (1.8)	32.1 (1.8)	27.5 (1.8)	27.5 (1.8)	26.7 (1.8)	27.0 (2.0)	29.0 (0.8)
New Brunswick	29.6 (1.7)	28.0 (1.7)	25.0 (1.7)	20.6 (1.7)	22.1 (1.7)	20.5 (1.9)	24.6 (0.7)
Quebec	31.1 (1.0)	29.3 (1.0)	25.5 (1.0)	23.8 (1.0)	24.0 (1.0)	24.6 (1.1)	26.6 (0.4)
Ontario	27.8 (0.8)	27.0 (0.9)	23.3 (0.9)	21.6 (0.8)	21.3 (0.9)	19.1 (0.9)	23.6 (0.4)
Manitoba	29.4 (1.7)	25.0 (1.6)	21.1 (1.6)	21.8 (1.7)	20.7 (1.7)	18.5 (1.8)	23.1 (0.7)
Saskatchewan	29.3 (1.7)	30.8 (1.8)	25.0 (1.8)	25.0 (1.8)	22.4 (1.8)	22.3 (2.0)	26.2 (0.8)
Alberta	29.0 (1.4)	29.3 (1.4)	25.5 (1.3)	24.0 (1.3)	21.1 (1.3)	21.3 (1.3)	25.2 (0.6)
British Columbia	23.8 (1.3)	21.8 (1.3)	21.0 (1.3)	19.0 (1.3)	18.6 (1.3)	19.3 (1.5)	20.7 (0.6)
Married	26.3 (0.6)	24.5 (0.5)	20.9 (0.5)	20.5 (0.5)	20.0 (0.5)	19.3 (0.5)	26.3 (0.5)
Separated	44.7 (1.3)	41.8 (1.4)	37.5 (1.4)	35.0 (1.4)	34.1 (1.4)	35.1 (1.6)	44.8 (1.4)
Single	28.0 (0.7)	28.1 (0.7)	25.0 (0.8)	28.1 (0.9)	26.7 (0.9)	22.8 (0.9)	28.0 (0.7)
Low income	40.0 (1.3)	42.0 (1.6)	41.8 (1.7)	44.4 (2.0)	39.5 (2.3)	41.2 (2.7)	41.3 (0.7)
Middle income	30.1 (0.6)	29.6 (0.6)	26.4 (0.6)	25.4 (0.6)	25.7 (0.7)	26.0 (0.8)	27.6 (0.3)
High income	20.7 (0.8)	22.0 (0.8)	19.5 (0.7)	19.0 (0.7)	18.4 (0.6)	18.1 (0.6)	19.5 (0.3)

The average smoking prevalence by selected groups was obtained from Canada National Population Health Survey (NPHS) 1998/99, 2000/01, 2002/03, 2004/05, 2006/07, & 2008/09. The statistics are weighted using the NPHS sampling weights. Standard errors are given in parenthesis.

**Table 4.** Smoking participation responses to cigarette taxes by gender, income level and health status.

	**Gender**		**Income level**		**Health status**	
	**Male**	**Female**	**Low**	**High**	**Low**	**High**
**Cigarette taxes**	−0.0082 *** (0.0022)	−0.0028 (0.0021)	−0.0051 ** (0.0022)	−0.0043 (0.0030)	−0.0051 * (0.0028)	−0.0073 *** (0.0021)
**APE**	−0.0025 *** (0.0007)	−0.0008 (0.0005)	−0.0017 *** (0.0007)	−0.0011 (0.0008)	−0.0017 * (0.0009)	−0.0020 *** (0.0006)
**Tax elasticity**	−0.3216 *** (0.0864)	−0.1198 (0.0881)	−0.1829 ** (0.0781)	−0.2017 (0.1381)	−0.1913 * (0.1038)	−0.3168 *** (0.0932)
***N***	26709	30061	32076	18153	21013	35723

*** Significance at 1% level,

** significance at 5% level, and

* significant at 10% level. Robust standard errors clustered at the individual level are in brackets. APE is the average partial effect. All results are population weighted. Low income category represents individuals in the low/middle income household and high income group are individuals in high income household. Low health individuals are those who report good/fair/poor health and high health for those who report excellent/very-good health.

**Table 5.** Smoking participation response to cigarette taxes by education.

	**Four-groups**				**Two-groups**	
	**Less secondary**	**Secondary**	**Some post secondary**	**Post secondary**	**Low**	**High**
Cigarette taxes	−0.0135 *** (0.0036)	−0.0060 (0.0040)	−0.0005 (0.0031)	−0.0010 (0.0025)	−0.0106 *** (0.0026)	−0.0008 (0.0019)
APE	−0.0038 *** (0.010)	−0.0020 (0.0013)	−0.0002 (0.0010)	−0.0003 (0.0007)	−0.0032 *** (0.0008)	−0.0002 (0.0006)
Tax elasticity	−0.5549 *** (0.1484)	−0.2179 (0.1445)	−0.0182 (0.1176)	−0.0422 (0.1079)	−0.4135 *** (0.1029)	−0.0332 (0.0777)
*N*	12807	7047	14726	20937	19854	35663

*** Significance at 1% level,

** significance at 5% level, and

* significant at 10% level. Robust standard errors clustered at the individual level are in brackets. APE is the average partial effect. All results are population weighted. Low education represents individuals with secondary education or less (high school or less) and high education is defined otherwise (greater than high school).

**Table 6.** Smoking participation response to cigarette taxes by age groups (1998–2008).

	**Three-groups**			**Two-groups**	
	**12–24**	**25–44**	**45–65**	**18–40**	**41–65**
Cigarette taxes	−0.0026 (0.0043)	−0.0031 (0.0026)	−0.0055 ** (0.0023)	−0.0004 (0.0025)	−0.0069 *** (0.0020)
APE	−0.0007 (0.0011)	−0.0010 (0.0008)	−0.0015 ** (0.0006)	−0.0001 (0.0008)	−0.0020 *** (0.0006)
Tax elasticity	−0.1217 (0.1994)	−0.1139 (0.0960)	−0.2404 ** (0.0995)	−0.0153 (0.0894)	−0.2955 *** (0.0837)
*N*	10910		22707	23529	28246

*** Significance at 1% level,

** significance at 5% level, and

* significant at 10% level. Robust Standard errors clustered at the individual level are in brackets. APE is the average partial effect. All results are population weighted.

**Table 7.** Smoking participation response to cigarette taxes by age groups (1998–2002).

	**Three-groups**			**Two-groups**	
	**12–24**	**25–44**	**45–65**	**18–40**	**41–65**
Cigarette taxes	−0.0186 ** (0.0081)	−0.0012 (0.0047)	−0.0122 ** (0.0052)	−0.0066 (0.0048)	−0.0113 ** (0.0044)
APE	−0.0050 ** (0.0022)	−0.0004 (0.0015)	−0.0035 ** (0.0015)	−0.0022 (0.0016)	−0.0033 ** (0.0013)
Tax elasticity	−0.6576 ** (0.2854)	−0.0352 (0.1350)	−0.4028 ** (0.1714)	−0.1830 (0.1332)	−0.3697 ** (0.1448)
*N*	6151	13592	11322	13926	14349

*** Significance at 1% level,

** significance at 5% level, and

* significant at 10% level. Robust standard errors clustered at the individual level are in brackets. APE is the average partial effect. All results are population weighted.

## Appendix

**Table A1.** Variables definition.

**Variable**	**Definition**
Smoking	=1 if currently a daily or occasional smoker, 0 otherwise
Cigarette tax	=Real excise cigarette tax per carton
Trend (*𝒯*)	=Linear year trend
Age	=age of respondents in years
Low_income	=1 if household income is low income group, 0 otherwise
Mid_income	=1 if household income is middle income group, 0 otherwise
High_income	=1 if household income in high income group, 0 otherwise
Male	=1 if gender is male, 0 otherwise
Female	=1 if gender is female, 0 otherwise
Household size	=Number of people living in an household
Employed	=1 if currently working, 0 otherwise
Unemployed	=1 if currently not working, 0 otherwise
Unemployment rate	=annual average unemployment rate by province of residence
Less_secondary	=1 if education is less than secondary, 0 otherwise
Secondary	=1 if education is secondary, 0 otherwise
Some post secondary	=1 if education is some post secondary, 0 otherwise
Post secondary	=1 if education is post secondary, 0 otherwise
Married	=1 if married/ living with a partner/common-law, 0 otherwise
Separated	=1 if widowed/separated/divorced, 0 otherwise
Single	=1 if never married, 0 otherwise (base category)
Canada	=1 if country of birth is Canada, 0 otherwise
Immigrant	=1 if country of birth is not Canada, 0 otherwise
Newfoundland	=1 if province of residence is Newfoundland, 0 otherwise
Prince Edward	=1 if province of residence is Prince Edward, 0 otherwise
Nova Scotia	=1 if province of residence is Nova Scotia, 0 otherwise
New Brunswick	=1 if province of residence is New Brunswick, 0 otherwise
Quebec	=1 if province of residence is Quebec, 0 otherwise
Ontario	=1 if province of residence is Ontario, 0 otherwise
Manitoba	=1 if province of residence is Manitoba, 0 otherwise
Saskatchewan	=1 if province of residence is Saskatchewan, 0 otherwise
Alberta	=1 if province of residence is Alberta, 0 otherwise
British Columbia	=1 if province of residence is British Columbia, 0 otherwise
**χ̂(*within individual means*)**	
Mhse_size	= within individual mean of household size variable
Mtrend	= within individual mean of aggregate year trend
Mmarried	= within individual mean of ‘married’ variable
Mseparated	= within individual mean of ‘separated’ variable
Memployed	= within individual mean of ‘employed’ variable
Munemployment	= within individual mean of ‘unemployment’ variable

**Table A2.** Estimation results for smoking participation for the overall sample, aged 12–65.

	**Correlated Probit Model**			
	Coef.	S.E.	APE	S.E.
Cigarette tax	−0.005 ***	(0.001)	−0.002 ***	(0.000)
Trend (T)	0.023	(0.005)	0.001	(0.001)
Age	−0.007 ***	(0.001)	−0.002***	(0.000)
Newfoundland	0.311	(0.197)	0.100	(0.068)
Prince Edward	0.279 **	(0.132)	0.089 **	(0.045)
Nova Scotia	0.226 ***	(0.083)	0.071 ***	(0.027)
New Brunswick	0.085	(0.095)	0.026	(0.030)
Quebec	0.132 *	(0.068)	0.040 *	(0.021)
Ontario	0.060	(0.056)	0.018	(0.016)
Manitoba	–0.016	(0.083)	–0.005	(0.024)
Saskatchewan	0.067	(0.080)	0.020	(0.024)
Alberta	0.070	(0.072)	0.021	(0.022)
Male	0.115 ***	(0.030)	0.034 ***	(0.009)
Married	0.145 ***	(0.041)	0.042 ***	(0.012)
Separated	0.245 ***	(0.053)	0.077 ***	(0.017)
Secondary	0.183 ***	(0.052)	0.056 ***	(0.017)
Some post secondary	0.104 ***	(0.040)	0.031 ***	(0.012)
Post secondary	−0.134 ***	(0.041)	−0.039 ***	(0.012)
Mid_income	−0.041	(0.026)	−0.012	(0.007)
High_income	−0.235 ***	(0.032)	−0.068 ***	(0.009)
Employed	0.322 ***	(0.022)	0.091 ***	(0.006)
Unemployment rate	0.002	(0.008)	0.000	(0.003)
Household size	−0.037 ***	(0.010)	−0.011 ***	(0.003)
Immigrant	−0.280 ***	(0.051)	−0.077 ***	(0.013)
Mhse_size	−0.014 ***	(0.019)	−0.027 ***	(0.005)
Mtrend	−0.067 ***	(0.013)	−0.019 ***	(0.004)
Mmarried	−0.079	(0.069)	−0.023	(0.021)
Mseparated	0.276 ***	(0.097)	0.0821 ***	(0.028)
Memployed	−0.047	(0.052)	−0.014	(0.015)
Munemployment	−0.024	(0.018)	−0.007	(0.005)
Tax elasticity	−0.227 ***	(0.062)		
*N*	56770			

*** Significance at 1% level,

** significance at 5% level, and

* significant at 10% level. S.E is robust standard errors clustered at the individual level. APE is the average partial effect. All results are population weighted.
